# Shelf Life Evaluation of Clinical Grade Chondrogenic Induced Aged Adult Stem Cells for Cartilage Regeneration

**DOI:** 10.1038/s41598-018-22748-1

**Published:** 2018-03-12

**Authors:** C. C. Ude, W. T. Seet, S. Sharen Aini, B. S. Aminuddin, B. H. I. Ruszymah

**Affiliations:** 1 Tissue Engineering Centre, Universiti Kebangsaan Malaysia Medical Centre, Jalan Yaacob Latif, Bandar Tun Razak, Cheras, 56000 K. L Malaysia; 2 Bioartificial Organ and Regenerative Medicine Unit, National Defence University of Malaysia, Sungai Besi, Camp, 57000 K. L Malaysia; 3 ENT Consultant Clinic, Ampang Putri Specialist Hospital, 68000 Ampang, Malaysia; 4 Department of Physiology, Medical Faculty Universiti Kebangsaan Malaysia, Jalan Yaacob Latif, Bandar Tun Razak, Cheras, 56000 K. L Malaysia

## Abstract

The study objectives include, enhancing the proliferations of aged bone marrow stem cells (BMSCs) and adipose stem cells (ADSCs); and evaluating the shelf lives of clinical grade chondrogenically induced cells from both samples. ADSCs and BMSCs from 56 patients (76 ± 8 yrs) were proliferated using basal medium (FD) and at (5, 10, 15, 20 and 25) ng/ml of basal fibroblast growth factor (bFGF). They were induced to chondrogenic lineage and stored for more than 120 hrs in FD, serum, Dulbecco’s phosphate buffered saline (DPBS) and saline at 4 °C. In FD, cells stagnated and BMSCs’ population doubling time (PDT) was 137 ± 30 hrs, while ADSCs’ was 129.7 ± 40 hrs. bFGF caused PDT’s decrease to 24.5 ± 5.8 hrs in BMSCs and 22.0 ± 6.5 hrs in ADSCs (p = 0.0001). Both cells were positive to stem cell markers before inductions and thereafter, expressed significantly high chondrogenic genes (p = 0.0001). On shelf life, both cells maintained viabilities and counts above 70% in FD and serum after 120 hrs. BMSCs’ viabilities in DPBS fell below 70% after 96 hrs and saline after 72 hrs. ADSCs’ viability fell below 70% in DPBS after 24 hrs and saline within 24 hrs. Concentrations between 20 ng/ml bFGF is ideal for aged adult cells’ proliferation and delivery time of induced BMSCs and ADSCs can be 120 hrs in 4 °C serum.

## Introduction

It has been proposed that cell based therapy could be the ideal treatment for cartilage regeneration in osteoarthritis^1,2^. Early reports include using multipotent adult mesenchymal stromal cells (MSCs) specifically, bone marrow stem cells (BMSCs) and adipose derived stromal cells (ADSCs)^3–5^. These procedures did not only provide alternative cell candidates, but also allow manipulations to suitable cells for implantation^6–8^.

Several clinical trials and treatments have been initiated by different groups, mostly with minimally manipulated cells^9–11^. Allogeneic treatments have been given in lieu of autologous cells^12^, due to difficulty in obtaining and propagating cells from very old patients, as some ailments lie within the upper quartile of life. For instance, osteoarthritis (OA) is an age related ailment and has been reported to increase to 23% in persons over 55 years of age and 39% in those over 65 years^13^. Presently, most imported cells are stored at 4 degrees Celsius, for several hours before delivery and implantation. Most often, normal saline is the dissociation fluid and virtually all clinics do not repeat cell counts and viability estimation of imported cells prior to implantation.

Previously we reported cartilage tissue regeneration through chondrogenesis and i*n vivo* implantation of autologous chondrogenic induced BMSCs and ADSCs in OA sheep model. We showed that the implanted induced BMSCs and ADSCs repaired damaged articular cartilages and regenerated adjacent meniscus^7,8,14–16^. With the above results, there was the need to pursue a clinical trial.

As a prerequisite, the procedure of chondrogenic induction has to be optimised with clinical grade reagents in a GMP facility^17^. Adequate quality controls and validation of product release criteria are pertinent to standard operation procedures^18^. Above all, the optimum viability of final products at delivery time and implantation is required to guarantee safety, efficacy and best outcome. Hence our objective in this study is to boost the proliferative ability of aged BMSCs and ADSCs; and evaluate the shelf life of clinical grade autologous chondrogenic induced BMSCs and ADSCs after storage at +4 degrees Celsius.

## Methods

### Experimental Design

Ethics approval was granted by the National University of Malaysia Medical Research and Ethics Committee (Code: UKM 1.5.3.5/244/PRGS/1/13/SG06/UKM/01/1), in compliance with the International Conference of Harmonization (ICH), Good Clinical Practice Guidelines. Written informed consent, approved by the committee was obtained from all participants. A total of 56 patients from both sexes, aged 76 ± 8 yrs were involved. Exclusion criteria include patients with infected joints, active malignancies, positive retroviral status, hepatitis A and B. They were divided into BMSCs and ADSCs groups. Bone marrow was collected from 30 patients undergoing total knee replacement or joint reparation surgery. Adipose tissues were obtained from 26 patients undergoing liposuction or other procedures. Stem cells were isolated and cultured in all clinical grade media, containing pooled human serum. They were proliferated at several concentrations of bFGF, targeting an optimized injectable number of 2 × 10^7^ cells. Growth kinetics was done and both cells were induced to chondrogenic lineage for 3 weeks. Chondrogenic genes were assessed. Cells were aliquoted at a concentration of 5 × 10^5^ cells into 5 different 15 ml tubes containing different media and stored at +4 °C. Thereafter, histology, immunohistochemistry, cell counts and viabilities were done.

### Sample Collection

Redundant bone marrow was collected from patients during total knee replacement or joint repairment surgeries. A Trocar (Cardinal Health Inc. USA) was inserted directly at the exposed distal end of femur or the proximal end of tibia. With the aid of a 50 ml syringe (Cringe^TM^ Malaysia) containing 0.5 mL of 10^3^ IU heparin, 8 ± 2 mls of bone marrow was aspirated and kept at +4 °C until further processing. Adipose tissue was obtained, using clinical grade disposables, from liposuction and other discarded tissues from trauma and accidents. About 10 g of adipose tissue was utilized in a given sample.

### Cell Isolation and Culture

BMSCs were isolated via gradient centrifugation over a Ficoll-Paque PLUS layer (Sigma-Aldrich), at 1008 g for 30 min and subsequently washed twice with phosphate buffered saline (PBS) (Gibco USA). Adipose tissue was minced to thickness of about 2 mm in Petri dish, before digestion with an equal volume of 0.6% collagenase type I. The digest was filtered with a 100 µm cell strainer. Filtrate was centrifuged at 2800 g for 5 mins at 37 °C. Pellet was washed with PBS and basal medium before culture. Cells were resuspended in basal culture medium FD (Ham’s F12: high glucose DMEM 1:1) (Sigma-Aldrich), supplemented with 10% human serum and incubated at 37 °C in a humidified atmosphere containing 5% CO^2^. Medium was changed every 2 to 3 days.

### Cell Viability and Growth Kinetics

Growth kinetics and viability were performed using trypan blue (0.4%, Sigma-Aldrich) exclusion assay with Neubauer haemocytometer. Population doubling time (PDT) was used to determine the growth of BMSCs and ADSCs. The addition of basic fibroblastic growth factor (bFGF) (Sigma-Aldrich) sequentially and in media constitution with five different concentrations (5, 10, 15, 20 and 25) ng/ml was explored for proliferation enhancement.

### Flow Cytometry

Cell characterization was done through immunophenotyping to determine if they express markers characteristic of mesenchymal stem cells. Both cells were incubated with antibodies tagged with fluochrome, each for 30 mins on ice. The positive markers to MSCs were (CD 44, CD 73, CD 90, CD 105) and those for the negative markers were (CD 10, CD 34, CD 45) (Sigma–Aldrich, USA). Cells were washed and resuspended with sheath fluid. Data were acquired using BD FACS Calibur™ flow cytometry system (Becton Dickinson, USA).

### Chondrogenic Inductions

BMSCs were induced to chondrogenic lineage with medium containing serum, ITS (GIBCO), ascorbic acid-2 phosphate, L-proline, dexamethasone, transforming growth factor beta 3 (TGF-β3), and insulin like growth factor 1(IGF-1)^16^. ADSCs were induced with a medium containing all the above components and BMP-6^19^. Uninduced BMSCs and ADSCs were the negative controls; while, primary chondrocytes were the positive. Medium was changed every 3 days and induction period lasted for 21 days.

### Gene Expression Analysis

Total RNA was extracted from uninduced BMSCs and ADSCs; chondrogenic-induced BMSCs and ADSCs; and the primary chondrocytes using conventional Trizol method. The expression of hyaline cartilage markers: aggrecan (*ACAN*), collagen type II alpha 1 (*COL2A1*), collagen type IX alpha 1 (*COL9A1*), sry (sex determining region y)-box 9 (*SOX9*), collagen type IX alpha 1 (*COLIXA1*) and fibromodullin (*FMOD*), together with the negative markers: collagen type I alpha 1 (*COL1A1*) and collagen type X alpha 1 (*COL10A1*) were evaluated by quantitative real-time PCR method (iScript™ One Step RT-PCR Kit with SYBR^®^ Green and iQ^TM^ SYBR^R^ green super mix (BIO-RAD). Expression level of each targeted gene was normalized to the housekeeping gene, human glyceraldehyde-3-phosphate dehydrogenase (*GAPDH*). This was performed at reaction profile of cDNA synthesis for 30 min at 50 °C, pre-denaturation for 2 min at 94 °C, PCR amplification for 38 cycles in 30s at 94 °C, 30s at 60 °C and 30s at 72 °C.

### Shelf-life Evaluation of Chondrogenic Induced BMSCs and ADSCs

Chondrogenic induced BMSCs and ADSCs were stored at 4 °C for (24, 48, 72, 96 and 120) hrs. At each point, evaluations of morphology, cell count, viability, proliferation and immunocytochemistry were done. A portion of the chondrogenic induced BMSCs and ADSCs were evaluated at day zero as controls. Cells were aliquoted in 5 different 15 ml tubes containing (FD, serum, DPBS and saline) at a concentration of 5 × 10^5^ cells per tube. They were stored at +4 °C and one tube was evaluated per day. Afterwards, the remnant content of the tube was cultured for morphology, proliferation and immune assay. Data were compared with records of day zero samples.

### Histological Evaluations

Cells were fixed for 1 hour with 4% paraformaldehyde (Sigma-Aldrich)prior to toluidine blue and safranin O staining, to assess cartilage proteoglycans and glycosaminoglycan deposition respectively^16^. These were evaluated by three blinded scorers, using the validated Simple Histological Histochemical scoring system (Modified O’ Driscoll), that is based on cellular morphology and the uptake of toluidine blue and safranin O stain. It has four grade scales, 0–3. A score of 0 means almost no cartilage and a score of 3 means almost all cartilage^20^.

### Immunocytochemistry

COL1A1, COL2A1, ACAN, and COL10A1 were evaluated. Fixed cells were permeabilized for 5 minutes with 0.1% Triton X-100 solution (Sigma-Aldrich) and blocked with 10% goat serum (Sigma-Aldrich) for 30 minutes at 37 °C. Monoclonal primary antibodies (mouse anti-human collagen type 1 alpha 1, collagen type 2 alpha 1, aggrecan and collagen type 10 alpha 1) (Sigma–Aldrich) were diluted into 1:200 parts. Samples were incubated with these antibodies overnight at +4 °C. After washing off primary antibodies, cells were incubated with Alexa Fluor® 594 goat anti-mouse (red-fluorescent dye) (Invitrogen USA) and Alexa Fluor® 488 goat anti-mouse (green-fluorescent dye) (Invitrogen USA) for 1 hr, at 37 °C and further counterstained with DAPI (Dako) for 15 minutes. Evaluation was done using a Nikon Eclipse Ti confocal microscope (Nikon, Tokyo).

### Statistical Analysis

Data were presented as mean ± standard deviation (SD) of sample size. The parametric means of cell counts, population doubling time and viabilities were analysed using paired t-tests. The histology quantification and gene expressions were done using the one-way ANOVA. Both were further analysed with Bonferroni’s post-hoc multiple comparisons for all pairs. Uncertainties were presented within 95% confidence intervals and all statistical analysis was performed using the version 17.0 SPSS software and the graph pad prism5.

### Data Availability

The dataset generated during and/ or analysed during then current study are available from the corresponding author on reasonable request.

## Results

### Cell Growth Kinetics Evaluations

**Figure 1:**
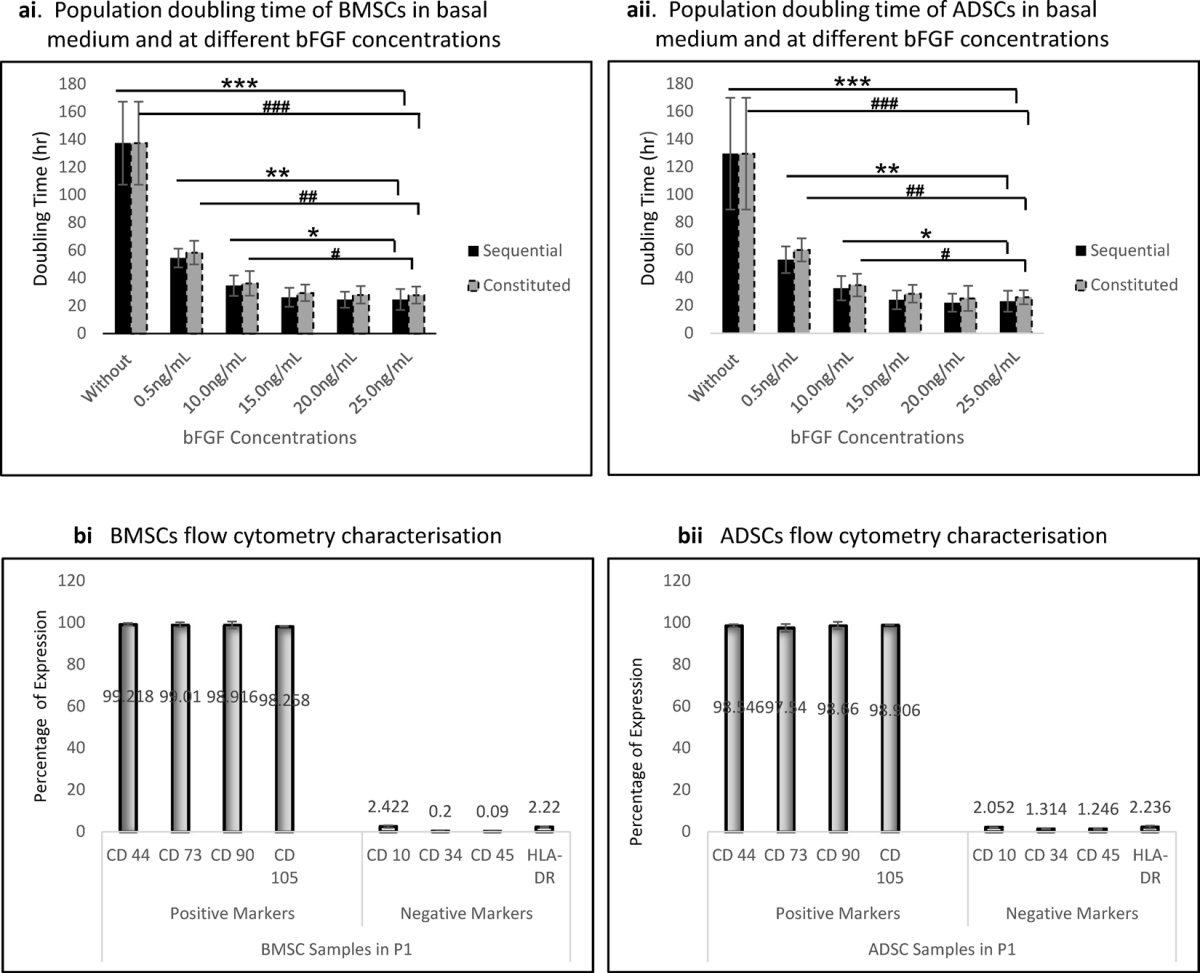
Growth Kinetics and Monolayer phenotype characterisation of cell samples. (**ai**) Population doubling time of BMSCs in basal medium and at different concentrations of bFGF. PDT without bFGF was significantly higher compare to every addition of bFGF, (***)p = 0.0001. 5 ng/ml was significantly higher to other concentrations in both sequential and constitution addition, (***)p = 0.0001. The same was found at 10 ng/ml, (*)p = 0.005. At 15, 20 and 25 ng/ml, there was no significance difference. (**aii**) Population doubling time of ADSCs at different concentrations of bFGF. Without addition of bFGF, PDT was significantly higher to every addition of bFGF, (***)p = 0.0001. PDT at 5 ng/ml was significantly higher to other increased concentrations (***)p = 0.0001. The same at 10  ng/ml, (*)p = 0.005. There was no significance difference between PDTs at (15, 20 and 25) ng/ml. (**bi**) BMSCs flow cytometry. BMSCs were highly positive to the markers CD 44, CD73, CD90 and CD105 associated with positive markers of stem cells. They were also negative to CD 10, CD34, CD45 and HLA-DR associated with negative markers of stem cells. (**bii**) ADSCs flow cytometry. ADSCs were highly negative to CD 44, CD73, CD90 and CD105 associated with positive markers of stem cells. They were also negative to CD 10, CD34, CD45 and HLA-DR associated with negative markers of stem cells respectively.

FD medium only caused cells to stagnate, became hypertrophic, with multiple pseudopodia. BMSCs’ PDT was 137.5 ± 30.0 hrs, but following the addition of bFGF in sequence and in constitution with media, cells revived and PDT increased gradually with each concentration and plateaued at (20–25) ng/ml (Fig. 1ai). PDT without the addition of bFGF was significantly higher compare to every single addition of bFGF (***)p = 0.0001. PDT at 5 ng/ml was significantly higher than other increased concentrations in both sequential addition and constitution with media, (***)p = 0.0001. The same trend was found in 10 ng/ml, (*)p = 0.005. There was no significant difference between PDTs at (15, 20 and 25) ng/ml for both sequential addition and media constitution (Fig. 1ai). ADSCs’ PDT was 129.7 ± 40.3 hrs, when cells were cultured in FD medium only. Upon the addition of bFGF, sequentially or in constitution with media, the same trend of proliferation reported for BMSCs was observed at every concentration (Fig. 1aii). Note, that the addition of bFGF in sequence at each concentration, though not statistically significance, had reduced PDTs on each cell sample. PDTs at (20 and 25) ng/ml were almost equal in both cells (Fig. 1ai and aii)

### Characterization via Flow Cytometry

Both cells were highly positive for all positive markers of MSCs evaluated; and as well negative for all negative markers. BMSCs had (99.22 ± 0.6, 99.01 ± 1.2, 98.92 ± 1.6 and 98.26 ± 0.2)%, for CD 44, CD73, CD90 and CD105; and (2.42 ± 0.6, 0.2 ± 0.1, 0.09 ± 0.0 and 2.22 ± 0.4)%, for CD 10, CD34, CD45 and HLA-DR respectively, (Fig. 1bi). ADSCs had also (98.55 ± 0.7, 97.54 ± 1.9, 98.66 ± 1.7 and 98.91 ± 0.1)%, for CD 44, CD73, CD90, CD105; and (2.05 ± 0.6, 1.3 ± 0.14, 1.25 ± 0.3 and 2.24 ± 0.7)%, for CD 10, CD34, CD45 and HLA-DR, respectively (Fig. 1bii).

### Chondrogenic Inductions

**Figure 2:**
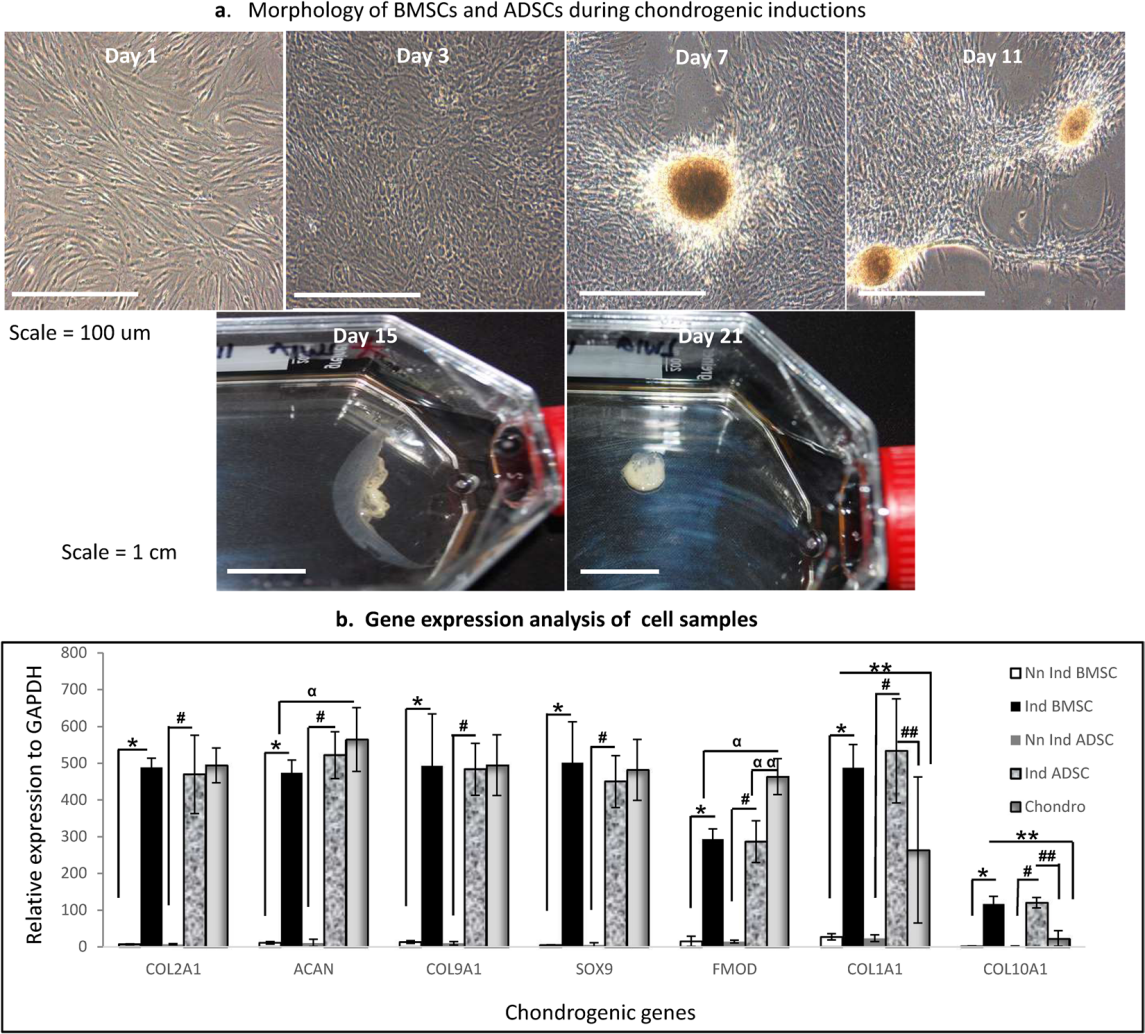
Chondrogenic Inductions. (**a**) Morphologies of BMSCs and ADSCs during chondrogenic inductions. The characteristic morphologies of both BMSCs and ADSCs as they underwent chondrogenesis were shown. The spindle morphologies of uninduced BMSCs and ADSCs (day 1), on interaction with induction media, changed to polygonal shapes (day 2), formed aggregates of cells and matrixes (days 7 to 11), and later become whitish cartilage structure (day 15 to 21) (**b**) RTPCR gene expression analysis. *COL2A1*, *ACAN*, *COL9A1*, *SOX9*, *FMOD*, *COL1A1* and *COL10A1*, revealed that chondrogenically induced BMSCs and ADSCs had significantly higher expressions compare to their uninduced forms (*, #)p = 0.0001. The native chondrocytes as positive control had increased expressions in all the hyaline genes and were significantly higher than induced BMSCs at *ACAN* and *FMOD* (^*a*^), and induced ADSCs at *FMOD* (^*a a*^)p = 0.005. Both BMSCs and ADSCs had significantly higher expressions compare to the native chondrocytes at *COL1A1* and *COL10A1* respectively (**, ##)p = 0.005.

The characteristic changes in cell morphologies as both BMSCs and ADSCs undergo chondrogenesis were shown. The spindle, fibroblastic morphologies of the uninduced BMSCs and ADSCs, upon interaction with induction media, formed aggregates of cells and matrixes that became whitish cartilage structure (Fig. 2a). The gene expressions of *COL2A1*, *ACAN*, *COL9A1*, *SOX9*, *FMOD* and the dedifferentiation markers *COL1A1* and *COL10A1*, revealed that chondrogenically induced BMSCs and ADSCs had significantly higher expression in all genes compared to the uninduced negative controls (*, #)p = 0.0001 (Fig. 2b). The native chondrocytes, as the positive control had increased expressions compared to induced BMSCs and induced ADSCs in all hyaline genes. They were significantly higher than induced BMSCs for *ACAN* and *FMOD* (^*a*^); and induced ADSCs for *FMOD* (^*a a*^)p = 0.005, (Fig. 2b). The dedifferentiation markers showed that both induced BMSCs and ADSCs had significantly higher expressions compared to native chondrocytes (**, ##) respectively, p = 0.005, (Fig. 2b).

### Shelf-life Evaluation

**Figure 3:**
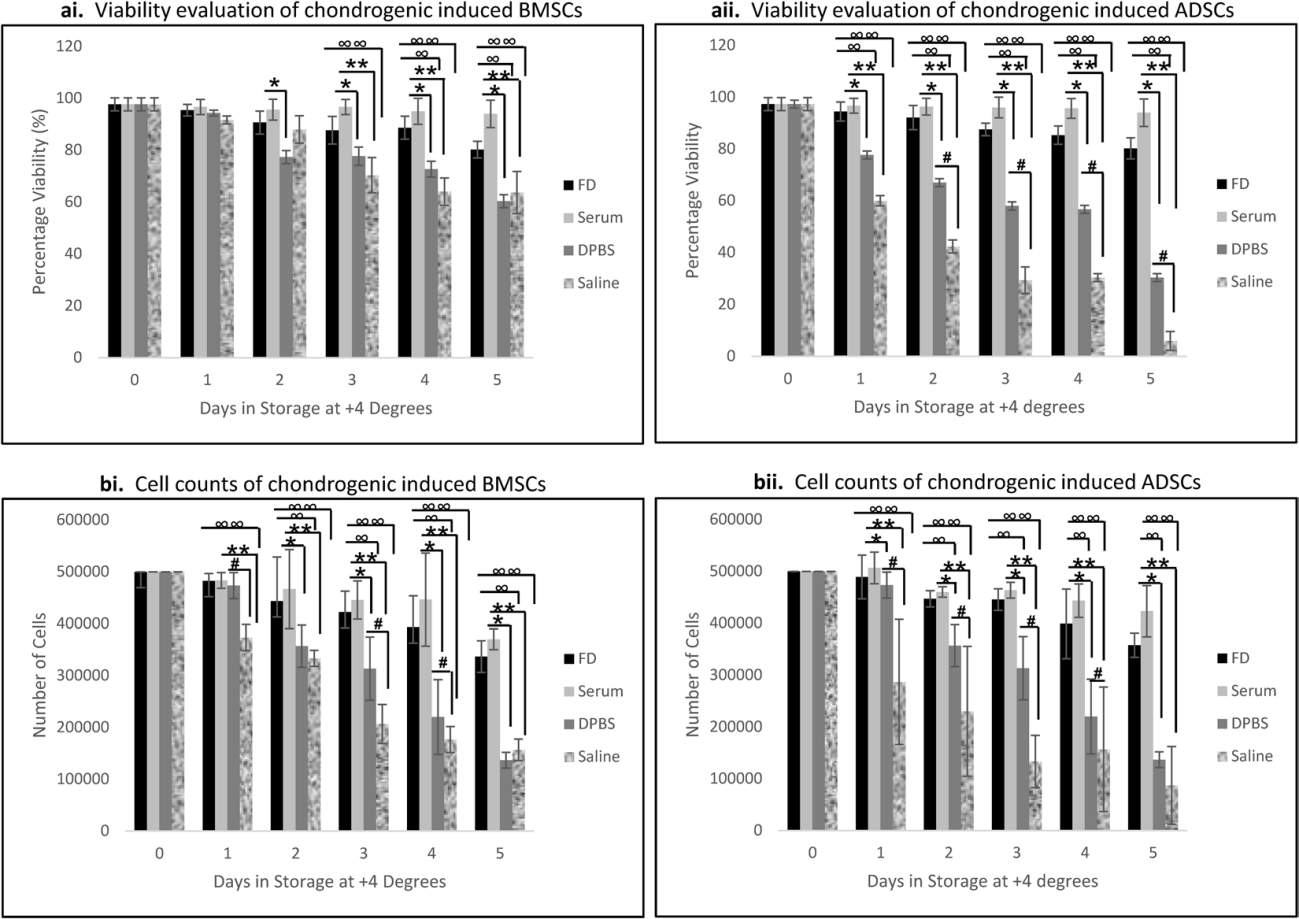
Shelf life evaluation of revived chondrogenic induced cell samples in different media (**ai**) Viability evaluation of chondrogenic induced BMSCs. On day 1, viability decreased slightly in all media, but was not significant. On the second day, viability of cells in DPBS and Saline decrease more. Serum had higher viability to DPBS only (*)p = 0.003. From the third day through fifth, further decreases occurred in DPBS and Saline. Serum and FD remained highly comparable with day 1 values. Both were significantly higher to DPBS and Saline (*, **) (∞, ∞∞)p = 0.001 respectively. (**aii**) Viability evaluation of chondrogenic induced ADSCs. From day 1, viabilities decreased drastically in both DPBS and Saline. FD and Serum maintained significantly higher values (*, **), (∞, ∞∞)p = 0.001. From the second day through fifth, more decreases were observed in DPBS and Saline. Both Serum and FD were significantly higher to DPBS and saline (*, **), (∞, ∞∞) respectively. DPBS also had significantly higher viabilities to saline (#)p = 0.0001. (**bi**) Cell counts of chondrogenic induced BMSCs. On day 1, a significant decrease was observed with Saline compare to FD, Serum and DPBS, (∞∞), (**), (#) respectively, P = 0.001. On the second day, FD and Serum remained significantly higher to DPBS and Saline (∞, ∞∞), (*, **) respectively, p = 0.0001. From the third and fourth day, counts in FD and Serum were significantly higher to both DPBS and Saline, while DPBS was also higher to Saline (∞, ∞∞), (*, **), (#) respectively, p = 0.0001. On the fifth day, FD and Serum remained significantly higher (∞, ∞∞) (*, **)p = 0.0001. (**bii**) Cell counts of chondrogenic induced ADSCs. There was a significant decrease with cells in DPBS and Saline compare to FD and Serum from day 1 (∞, ∞∞), (*, **)p = 0.001; DPBS also had significantly higher counts to saline (#)p = 0.001. The same trend was seen on days 2, 3 and 4 (∞, ∞∞), (*, **), (#)p = 0.001 respectively. On the fifth day, FD and Serum retained significantly higher difference to DPBS and Saline, but DPBS was not difference to Saline (∞, ∞∞), (*, **)p = 0.001 respectively.

Chondrogenic induced BMSCs had viabilities of approximately 100% at day zero in all the 4 media. On the first day of incubation, viability decreased slightly in all media, but was not significant. On the second day, viability of cells in FD and serum remained above 90%, but decrease occurred in DPBS and saline. Serum had higher viability than DPBS only (*)p = 0.003. From the third day, through the fifth, further decrease occurred in viabilities of cells in DPBS and saline. Serum remained highly comparable with day 1 values, while FD maintained viabilities above 80%. Both were significantly higher than DPBS and saline (*, **) (∞, ∞∞)p = 0.001 respectively, (Fig. 3ai).

The chondrogenic induced ADSCs’ viabilities were approximately 100% at day zero in all 4 media. From the first day, unlike BMSCs, there were drastic decrease in both DPBS and saline. FD and serum maintained significantly higher values (*, **), (∞, ∞∞)p = 0.001. From the second day through the fifth, severe decrease were observed in DPBS and saline. Serum remained above 90%, while FD maintained above 80%. Both were significantly higher than DPBS and saline (*, **), (∞, ∞∞) respectively. DPBS also had significantly higher viabilities than saline (#)p = 0.0001, (Fig. 3aii). Note that both cell samples maintained their viabilities up to 94% in serum at 5^th^ day.

Cell counts at day zero were 5 × 10^5^ cells in all media. For the induced BMSCs (Fig. 3bi), a significant decrease was observed from the first day on the cells in saline compare to FD, serum and DPBS, (∞∞), (**), (#) respectively, P = 0.001. On the second day, FD and serum retained significantly higher counts than DPBS and saline (∞, ∞∞), (*, **) respectively, p = 0.0001. From the third and fourth day, continuous cell reduction in DPBS and saline were recorded. Counts in FD and serum were significantly higher than both, while DPBS was higher than Saline (∞, ∞∞), (*, **), (#) respectively, p = 0.0001. On the fifth day, cell number reduced to 1.4 × 10^5^ in DPBS and 1.6 × 10^5^ in saline. FD and serum remained significantly higher (∞, ∞∞) (*, **)p = 0.0001.

Induced ADSCs (Fig. 3bii), had significant decrease on cell count for cells in DPBS and saline compared to FD and serum from day one (∞, ∞∞), (*, **)p = 0.001. DPBS had significantly higher counts than saline (#)p = 0.001. The same trend was observed on days 2, 3 and 4 respectively. On the fifth day, FD and serum retained cell counts up to 75% of day zero. DPBS had 1.4 × 10^5^ cells, while saline had only 8.7 × 10^4^ cells; FD and serum were significantly higher. Note that there was a marked decrease with the chondrogenic induced ADSCs counts in saline from the second day through the fifth day.

### Histological Evaluations of Cell Samples

**Figure 4:**
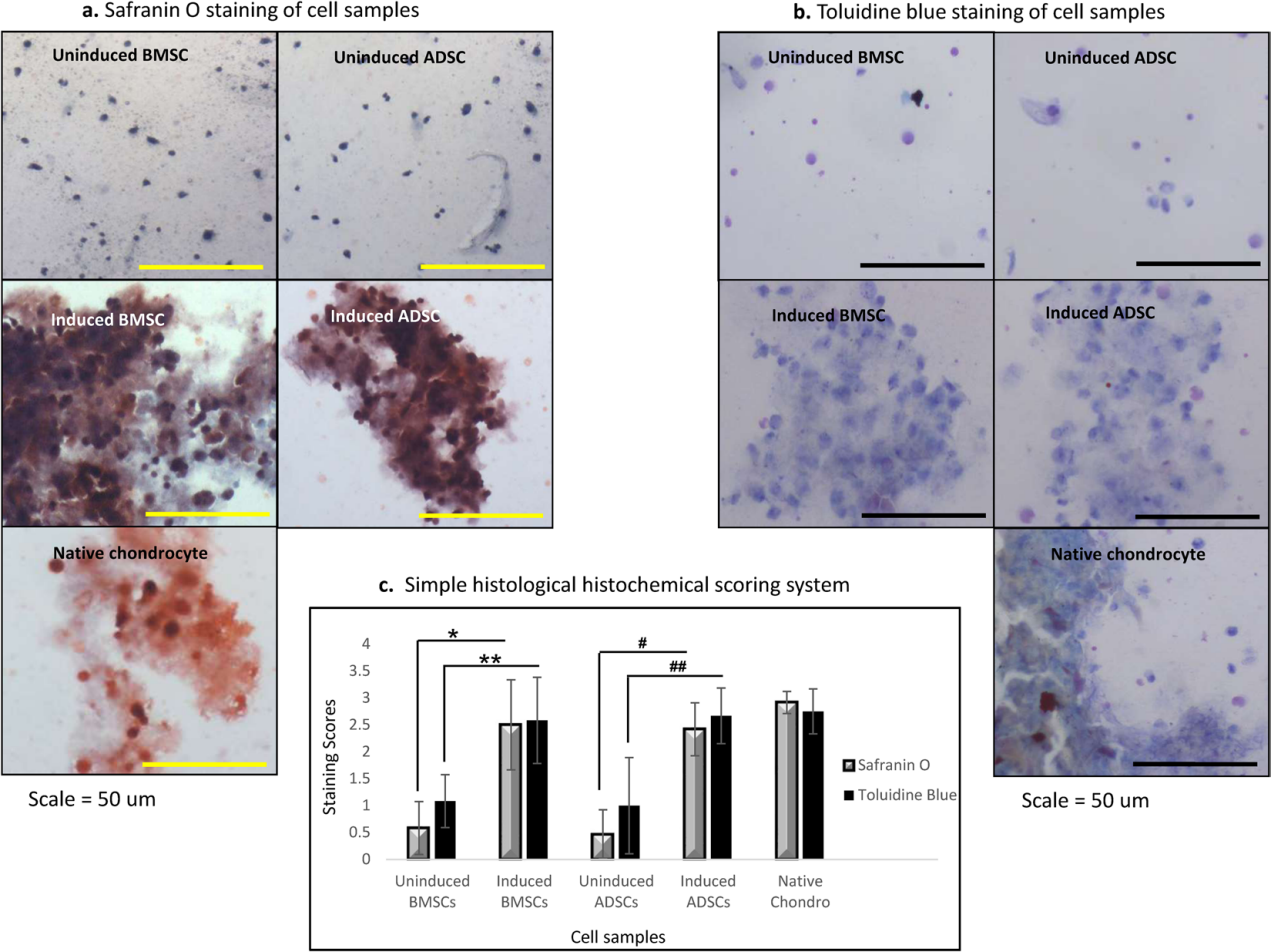
Histological evaluations of cell samples. (**a**) Safranin O staining of cell samples. Chondrogenic induced ADSCs and BMSCs stained positive. The appearances were in accordance with the presence of accumulated glycosaminoglycans. The two tests samples and the positive control (chondrocytes) were comparable, while uninduced cells were negative to the stain. (**b**) Toluidine blue staining of cell samples. Chondrogenic induced ADSCs and BMSCs stained positive. Cells and matrixes of test samples exhibited the presence of accumulated proteoglycans revealed via Toluidine blue. The two tests samples and the positive control (chondrocytes) were comparable, while uninduced cells were negative has slight positive stain. (**c**) The simple histological scoring Evaluation. Both chondrogenic induced BMSCs and ADSCs had significantly higher stains on both Safranin O and Toluidine blue compare to their negative control samples (*, **), (#, ##) respectively, p = 0.001. Both induced cells and the chondrocytes had no significant differences.

For both ADSCs and BMSCs, uninduced cell samples served as negative controls; chondrogenic induced cells were the test, while chondrocytes were the positive controls. On safranin O staining, induced ADSCs and BMSCs were positive with appearances in accordance with the presence of accumulated glycosaminoglycans revealed via safranin O staining. Little differences existed between the two tests samples compare to the positive control (Fig. 4a). The same quality of staining was observed with toluidine blue. Cells and matrixes of test samples exhibited the presence of accumulated proteoglycans revealed via toluidine blue (Fig. 4b). The simple histological scoring by three independent blinded observers revealed that both chondrogenic induced BMSCs and ADSCs had significantly higher stains compared to their negative control samples (*, **), (#, ##) respectively, p = 0.001. Both induced cells and the chondrocytes had no significant differences (Fig. 4c).

### Proliferation Evaluations of Stored Cells

**Figure 5:**
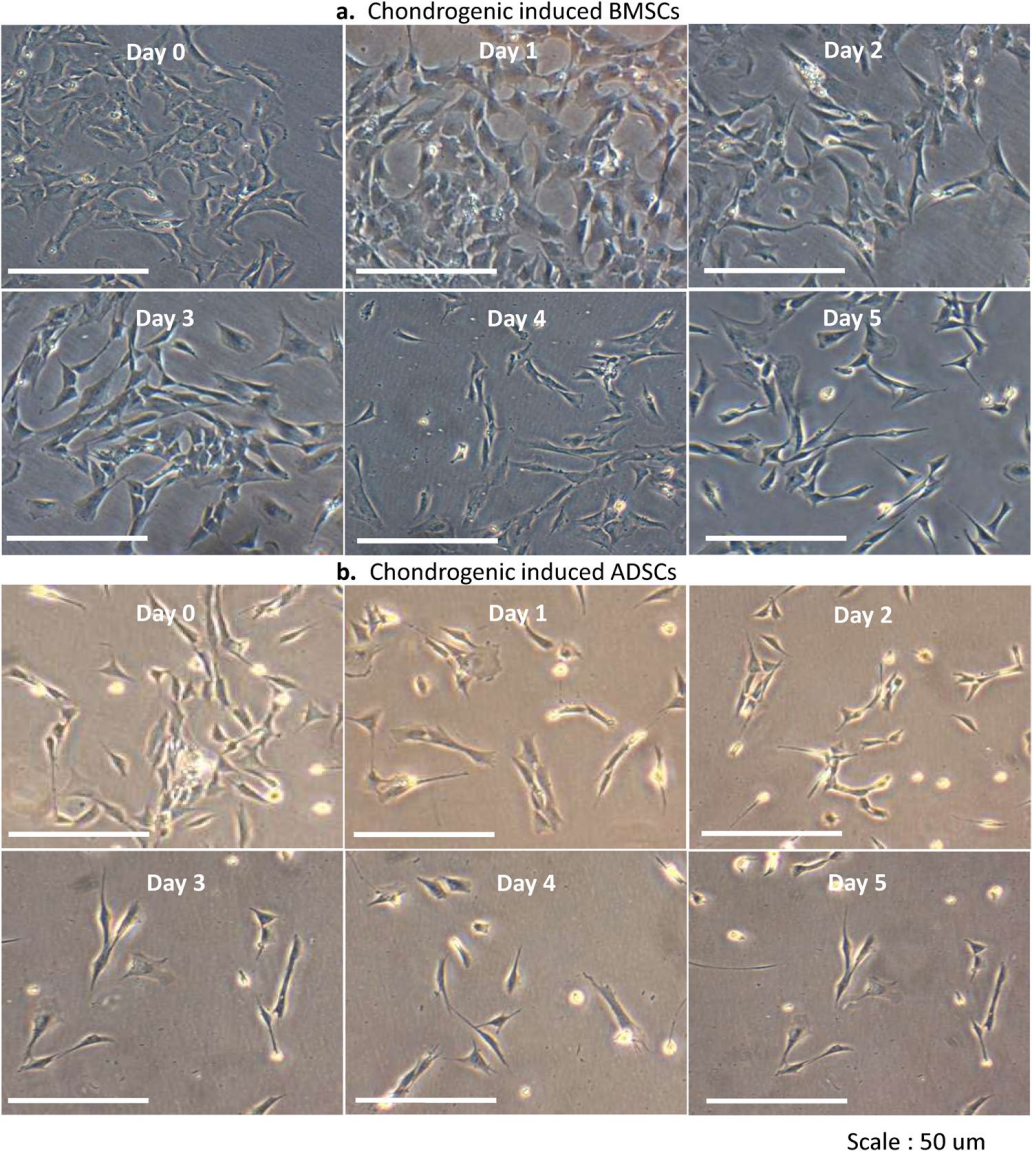
Attachment and proliferation of revived chondrogenic induced cell samples. (**a**) BMSCs. There were attachments and proliferations of the cells after storage. It provided evidence that the stored induced BMSCs were viable and proliferating. (**b**) ADSCs. There were attachments and proliferations of the cells after storage. It provided evidence that the stored induced ADSCs were viable and proliferating.

Induced BMSCs and ADSCs stored in serum were cultured back after each day’s analysis. This revealed attachments and proliferative ability of the cells after storage. It provided evidence that the cells were not technically viable, as was observed in some cases; but viable and proliferating (Fig. 5a and b).

### Immunocytochemistry

**Figure 6:**
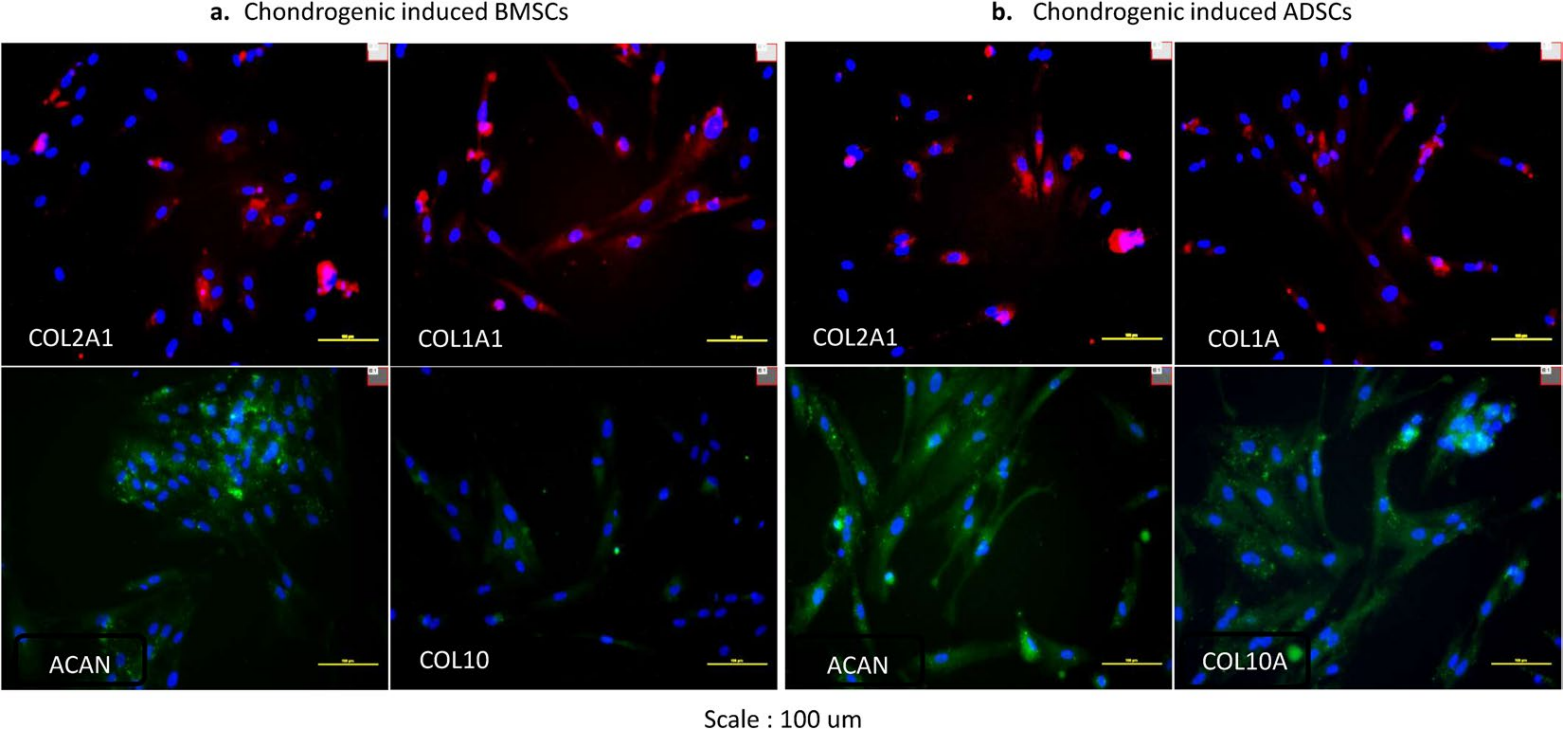
Immunohistochemistry of revived chondrogenic induced cell samples in serum (day 1). (**a**) BMSCs. The positive expression of collagen type I alpha 1, collagen type II alpha 1, aggrecan, and collagen type X alpha 1, evaluated on stored chondrogenic induced BMSCs in serum, on day 1 after cultures (**b**) ADSCs. The positive expression of collagen type I alpha 1, collagen type II alpha 1, aggrecan, and collagen type X alpha 1, evaluated on stored chondrogenic induced ADSCs in serum, on day 1 after cultures.

**Figure 7:**
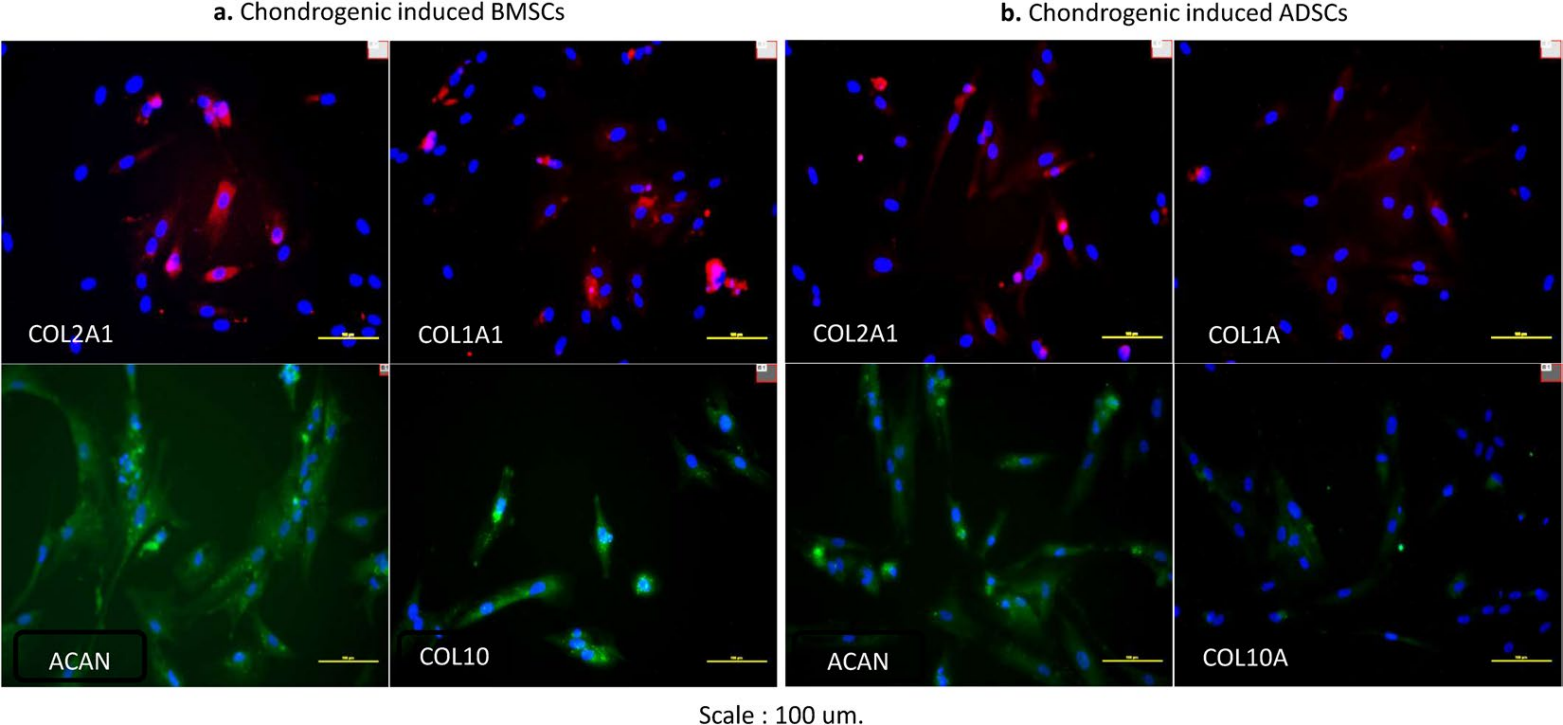
Immunohistochemistry of revived chondrogenic induced cell samples in serum (day 5). (**a**) BMSCs. The positive expression of collagen type I alpha 1, collagen type II alpha 1, aggrecan, and collagen type X alpha 1, evaluated on stored chondrogenic induced BMSCs in serum, on day 5 after cultures (**b**) ADSCs. The positive expression of collagen type I alpha 1, collagen type II alpha 1, aggrecan, and collagen type X alpha 1, evaluated on stored chondrogenic induced BMSCs in serum, on day 5 after cultures.

The expression of the chondrogenic proteins collagen type I alpha 1 (COL1A1), collagen type II alpha 1 (COL2A1), aggrecan (ACAN), and collagen type X alpha 1 (COL10A1) were evaluated on stored chondrogenic induced cells in serum, on the first and fifth day after cultures. (Fig. 6a and b) depicted the expressions of the above proteins in both cell samples at day 1. After the fifth day, the images in (Fig. 7a and b) also confirmed that the stored proliferating cells still retained their chondrogenic properties. The expression of COL1A1 and COL10A1 gave insight that all induced cells were not within the hyaline cartilage population.

## Discussion

To be ready for a clinical trial on cell therapy, quality product is obtained by following a predefined standard operation procedure^17,18^. From our earlier studies, 2 × 10^7^ autologous cells was effective in regeneration of osteoarthritic knee^8,16^. Owing to that, 2 × 10^7^ cells became our standard dose for a single injection. These cells should be within the 4^th^ passage and their viability must exceed 70% on production. Achieving this mark was easy with young animals induced to OA, as would be in young patients; but it is different in elderly patients with aged cells. Osteoarthritis lies within the prime age of life and autologous cell therapy is always desirable in order to avoid complications with tissue rejection^21^. Though stem cells are known to evade immune responses^22^, which makes allogeneic transplant an option, but in our case, because cells underwent major manipulation to potential final phenotype, it makes autologous therapy most suitable. This is because induced cells can express both major histocompatibility (MHC) class I molecules and MHC2, thus eliciting graft versus host disease^23^. From our best knowledge, this will be the first time a chondrogenically induced cells will be tried in clinics.

In proliferation of cells, it was observed that basic culture medium cannot aid the proliferation of aged BMSCs and ADSCs to reach the required number within the maximum time allowed, as PDT stood high at 138 hrs and 130 hrs respectively. Similar features have been reported in the past about ageing cells, owing to decrease telomerase activities^24^. We employed bFGF, a pro-angiogenesis factor, which is activated during wound healing^25^. It is also a critical component of human embryonic stem cell culture, and has been attributed to maintaining cells in an undifferentiated state^26^. Our results were significant with the least addition, 5 ng/ml of bFGF. It caused increased proliferation of BMSCs and ADSCs up to 20 ng/ml addition. Our evaluation on the sequential addition and constitution with media revealed that every sequential addition had better proliferating effect than its constitution equivalent. This may be as a result of immediate action on the cells against the constituting option, which might have been degraded by other components of the medium. Similar findings have been reported by Kim *et al*.^3^. ADSCs had slightly higher PDT at each concentration of bFGF, more than BMSCs as was reported earlier on the replications of ADSCs and BMSCs^16^. Following this results, sequential addition of 20 ng/ml bFGF was used as our standard to meet the required cell number within the allowed passage. Monolayer characteristics via flow cytometry revealed that both cell expressed positive markers of stem cells as we as the negative markers up to the 5^th^ passage. These were consistent with earlier reports^27^.

Chondrogenic induction with our specific cocktails proved encouraging. Both cells had significantly higher expressions compared to their uninduced cells on the gene evaluations. This was in accordance with earlier report^19^. They were comparable with the native chondrocyte except for *ACAN* and *FMOD* where the native was significantly higher; and for the dedifferentiation markers, where both induced cells had higher expressions. This was done to see the extent of fibrotic and osteogenic tilting of the induction; however, hyaline markers were highly expressed. Collagen type I alpha 1 is also expressed within the perichondrium of a normal hyaline cartilage and has been reported in 2D chondrogenesis^28^.

In real life situation, transplant products may require storage for certain period of time before grafting. Such delays as transportation, stabilizing patients’ conditions and facility, necessitate the need for evaluation of the quality of transplant organs, tissues or cellular products after storage, to guarantee safety and efficacy^18^. Hearts and lungs have shelf lives of 5 hours after harvest, liver has 18 hours; pancreas has 20 hours; kidney has 72 hours; cornea has 240 hours; heart valve, skin and bone has each 5 years^18^. Other cellular products like red cells have 42 days; paediatric red cells have 35 days; and washed red cells have 28 days, when stored between 2–6 °C^29,30^. Platelets have 5 days, when stored within 20–24 °C. Fresh frozen plasma, cryo depleted plasma and cryoprecipitate have shelf lives of 365 days at temperature below –25 °C^30,31^. It has been reported that some tissue engineered products may have shelf lives of 72 hours, beyond which they deteriorate in histological features as well as viability^32^. Considering these facts, we determined the shelf lives of chondrogenically induced BMSCs and ADSCs, when stored at +4 °C up to 120 hours in four different media (FD, serum, DPBS and saline). Serum retained the highest viability and total cell counts for both BMSCs and ADSCs throughout the storage duration. Serum was significantly higher than all others except FD, which retained the second highest viability and cell counts. It was interesting to note that saline which is the popular medium for dissociation and storage of most transported cellular products had the least of viabilities and cell counts. Though it has the advantage of direct injection to the body, over FD and DPBS; serum on the other hand is perfect with autologous therapy. It can also be used allogeneic as pooled human serum^33^.

Based on this outcome, other subsequent analysis were done with cells from serum at day 5. The histology images of the induced cells had similar pattern with the native chondrocytes revealing maturity. The proliferation of the induced cells confirmed viability and stability of cells. The immunocytochemistry of induced cells had similar expression with the native chondrocytes on the specific proteins assessed.

Furthermore, cells were stored up to 30 days in each media, exceeding our objectives. After day 25, while all cells were dead in DPBS and saline, a phenomenon we termed “technical viability” was observed in serum and FD media. In this, cells were viable by standards of trypan blue exclusion, but lost attachment and proliferation capability. This could be a topic for further study, as it might reshape how we judge viability of stored implant products; especially long term, as new study on blood cells has revealed that after 21 days, the membrane's integrity of stored blood cells degrades. This is a concern as most blood banks still transfuse red cells older than 3 weeks in storage^34^.

Among the limitations of this study include, the use of pooled human serum instead of autologous serum as intended. Considering the fact that, this was not the actual clinical trial, pooled serum were used as it was deemed unethical to aspirate blood from patients who would not benefit from the current cells and who were under medical treatments for other causes. The second limitation is the 2D culture, which is not ideal for chondrogenic induction. Being that we are pursuing an injectable cell therapy, we observed that most 2D cultured cells can pass through the syringe during transplant. In contrast, making 3D pellet cultures or seeding cells on scaffold will require further harvest and digestions; hence complicating the process and lowering viability.

## Conclusion

A sequential addition of 20 ng/ml of bFGF was most suitable in boosting the proliferation of aged adult cells and the delivery time for transplantation of chondrogenically induced BMSCs or ADSCs can be up to 120 hrs in serum at 4 °C.
